# Immigration and changes in the epidemiology of hemoglobin disorders in Italy : an emerging public health burden

**DOI:** 10.1186/1824-7288-38-32

**Published:** 2012-07-23

**Authors:** Francesco Cataldo

**Affiliations:** 1 Pediatric Department, University of Palermo, Past Secretary of Gruppo di Studio Gruppo di Lavoro Nazionale per il Bambino Immigrato della Società Italiana di Pediatria (GS GLNBI SIP), Palermo, Italy

**Keywords:** Immigration, Hemoglobinopathies

## Abstract

**Background:**

In the last years Italy is confronting with massive migratory movements from developing countries where hemoglobinopathies are widespread. This is causing a large diffusion and a changing spectrum in the epidemiology of hemoglobin disorders in Italy.

**Methods:**

Investigations recently published in Italy on hemoglobinopathies among immigrants were revised in order to appreciate the impact of immigration from developing countries on epidemiology of these pathologies and to outline adequate guidelines of prevention.

**Results:**

Although in Italy there is a limited number of investigations regarding the relation between immigration and hemoglobin disorders, published data show that in our Nation there is a changing and increasing spectrum of hemoglobinopathies linked to immigration.

**Conclusions:**

Prospective and retrospective actions of public healthy preventive policy are requested, based upon information (health educational programs for immigrants and caregivers), screenings among immigrants (school screening, pre-marital, preconception and early pregnancy screening, newborn screening), counseling for foreign at-risk couples and healthy carriers.

## 

The inherited disorders of hemoglobinopathies are among the most common monogenic diseases in the world, and about 4,5 % of all human beings carry a gene for these abnormalities [1,2]. Sickle Cell Disease (SCD) is by far the most common and is mainly widespread in Sub-Saharan Africa, Middle-East, India and in people of African ancestry living in Europe, North, Central and South America [3,4] . Thalassemias and hemoglobin E (HbE) are the second most common, occurring at a high frequency in Mediterranean area, Middle East, Central Asia, Indian Subcontinent, Southern China (β thalassemia) and in Middle East, Indian Sub Continent, South East Asia and parts of Mediterranean region (α thalassemia and HbE) [5,6]. Other less widespread anomalous hemoglobins are hemoglobin C (HbC) that is particularly common in West-Africa, and hemoglobin D (HbD) more frequent in South East Asia, Middle-East and Indian Sub Continent [4].Compound hemoglobins, (e.g. HbS/βthal, HbC/βthal, αThal/HbE, βThal/HbE, etc.) are relatively common, while other hemoglobinic anomalies clinically relevant are rare around the world [4]. It is believed that carriers of some hemoglobinopathies (α and β thalassemias, SCD, HbE) have a selective advantage in areas where malaria was endemic and that natural selection is responsible for elevating their gene frequencies [5,6].

Although hemoglobinopathies were rare in industrialized Northern and Central European countries, recently they have become much more common in these regions through the immigration from endemic areas [7-13]. So like during the first decades of the last century in North America thalassemia was firstly described in immigrant families from Southern Italy [14], recently Modell and co-workers [7] calculated that today in Europe more affected children are born in non historically endemic Northern and Central Nations than in Southern European Nations, due to migration [15,16]. Further, these rising frequencies of hemoglobinopathies in developed countries will likely increase in the next decades. Firstly because the immigration’s waves from poor regions of the world cannot be stopped and secondly because immigrant people are young and tend to get married in their same ethnic group and to have a high birth-rate, with a founder effect. In addition, immigrants have often consanguineous marriages which increase the frequency of all the Mendelian recessive disorders, and the majority of children affected by hemoglobinopathies in developed countries survive into adulthood because can receive optimal medical care. Therefore, nowadays hemoglobin disorders represent a significant health public health problem also in developing countries where precocious diagnosis of carriers or of affected individuals is crucial to enable the most appropriate actions of prevention and care.

Italy is historically a natural reservoir of hemoglobinopathies, especially β thalassemia and SCD, and more rarely also of HbC [2,9]. Nevertheless, in the last twenty years [17] there has been a rapid and progressive increase of immigration from developing countries where hemoglobinopathies are more frequent, particularly from Mediterranean area, Indian Sub-Continent and South-East Asia. Therefore, it should be considered that this immigration wave is likely to rise the frequency of hemoglobin variants in Italy. In our Nation we still have not an exact knowledge of both pathological hemoglobin’s carriers and hemoglobinopathies among immigrants, as well as of their epidemiological impact on the general resident population. Indeed, differently to other developed countries place of immigration [8,10-13,16,18,19], there are only few and limited investigations regarding this changing spectrum influenced by immigration. So, Garofalo and co-workers. [20]in Torino observed among 254 patients diagnosed between 1994 and 1998 as having hemoglobinopathies 13 immigrants (5.1 %), all Africans (10 with SCD and 3 with β thalassemia) and Furbetta and co-workers [21] among 962 residents in Umbria screened between 1987 and 1996 for hemoglobinopathies found 29 immigrants (3 %), mainly Africans (20 HbS, 4 βthal,2 HbA2 Babinga, 1 HbC and 2 Indians (HbE). According to these data, Russo-Mancuso and co-workers [22] in a clinical-epidemiological survey on SCD performed during 1998 in Italian pediatric and hematological Centers found 696 residents with this anomalous hemoglobin. 44 of them (6.3 %) had one or both foreign parents, mainly from Africa. Remarkably, these authors noted that HbS in the first years of their investigation had spread from Sicily and South Italy over all the Nation as a result of a domestic migration, whereas only in the last years immigration, mainly from Africa, had contributed to its large diffusion in our Nation.

Nevertheless, until a few years ago, there were not accurate studies regarding the impact of immigration from developing countries on epidemiology of hemoglobinopathies in Italy. Firstly Colombatti and co-workers [23] referred the pattern of hospitalization of patients aged < 15 years for SCD from 2000 to 2008 in the Veneto Region (North East Italy).They observed 135 SCD patients. 95 % of them were immigrants who were born abroad or in Italy from foreign parents, and their origin was mainly (more than 90 %) from Africa. A progressive increase in the number of inpatient admissions was observed in the studied period (55 in 2000, 94 in 2008).Successively (from January 2006 to May 2009) Lippi and co-workers [24]underwent a screening on 806 residents in Verona (Veneto Region) before marriage (432 Caucasians, 296 Africans and 78 Asian). 33/806 (4.1 %) hemoglobin variants were identified (HbS n = 20,HbC n = 5,HbE n = 3, Hb Lepore n = 1, HbD n = 1, other uncommon variant n = 3).Interestingly the frequency of HbS was remarkably high in Africans (19/296, 10 %), while HbE was confined in Asians(3/78, 4 %) and HbC was detected in 5/296 (1.7 %)Africans. The most accurate and comprehensive study on epidemiology of hemoglobinopathies among immigrants in Italy was carried out from Amato and co-workers [25,26]. They, from 1994 to 2007, performed a screening in secondary schools and to pregnant couples for hemoglobin variants in 167,235 residents in Lazio Region (Central Italy). 10,353 of the screened subjects were immigrants, with an increase during the studied period (2.7 % in 1994 to 9.7 % in 2007).2,484/10,353 immigrants (24 %) had anomalous hemoglobins, as carriers (n = 2,348, 94.5 %) or as affected (n = 136, 5.6 %). Among carriers 22.6 % were β Thal carriers, 48 % suspected α Thal carriers,13.8 % HbS carriers, 4 % HbE carriers and 1,8 % HbC carriers, while 4,3 % were carriers of other rare hemoglobins. The 136 affected patients mainly include SCD, β thalassemia major and intermedia, α thalassemia (hemoglobin H), homozygous HbC, homozygous HbE and compound heterozygous hemoglobinopathies . The ethnic groups and the respective more frequently associated hemoglobin variants observed both in carriers and in affected were : African (HbS n = 226, βThal n = 69, suspected αThal n =321),Asian (suspected αThal n = 283, βThal n = 92, HbE n = 90,HbS n =3),Central American (suspected αThal n = 136,HbS n = 55, βThal n = 46) and Central-East European (suspected αThal n = 333, βThal n = 280,HbS n = 18,HbE n = 3).

However, hemoglobin carriers and affected patients with foreign ancestry observed in Italy by the above mentioned investigations are those of subjects that have immigrated legally, whereas several unknown immigrants live illegally in Italy. Therefore, the number of foreign hemoglobin carriers and of immigrants affected by hemoglobinopathies is very large nowadays and likely it will increase in the next years. Consequently a public health policy and proper strategies of public health authorities are needed to avoid the rising frequencies of these disorders due to immigration, financing and facilitating prevention. Indeed financing prevention is economically sound when compared to the public health costs of treatment for a rapidly growing cumulative number of children with incurable hemoglobinopathies, who survive into adulthood. These preventive strategies of the public health authorities, that today are lacking in Italy, should be both prospective and retrospective, including three different types of actions : 1°) information for foreign populations and caregivers; 2°)healthy carriers detection among immigrants (screening programs); 3°) Counseling and prenatal diagnosis for healthy carriers and at-risk immigrant couples[16].

Information is the first step of prevention, and efforts should be made to reach each foreign groups, promoting healthy education programs. Notices on the hemoglobinopathies, translated in foreign languages and illustrated with figurative explanations, should be given by a physician who explains what they are and how they are prevented. This information have to provide knowledge, not anxiety or stigmatization, and needs to be adopted to the different cultures, paying attention to make clear that being a carrier is not a disease and that when a carrier is diagnosed family analysis is requested. At this regard, educational pre-matrimonial courses as well as scholastic information to adolescent pupils and 25 % possible recurrence risk can allow them to limit family size. In the same way, post academic courses on hemoglobinopathies should be organized for caregivers (general practitioners, pediatricians, obstetricians, midwifes)in order to improve their specific knowledge and their ability of communication with different cultures. x Prevention of a recessive genetic diseases, as the hemoglobinopathies, is usually associated with the concept of screening of healthy carriers by means of laboratory investigations. In the past the simple visual tube osmotic test and simple hemoglobin electrophoresis were systematically used as first step, while today separation of hemoglobin fractions on High Performance Liquid Chromatography (HPLC) is preferred. Molecular analysis, even if expensive and troublesome, must be always performed for prenatal diagnosis because it is essential for primary prevention. Indeed, a large and heterogeneous number of molecular mutations has been described for each hemoglobin variant [11,16].They are regionally/area specific (Mediterranean, Indian Asian, Southeast Asian, sub-Saharan Africa), and each region or area have a commoner but limited number of mutations together with a very large number of rare mutations that are associated with profound phenotypic diversity, not all the defects being equally severe. It is the case, for example, of the interaction HbE-βthalassemia in which patients with similar βthalassemia mutations may have different phenotypes (some with transfusions dependent anemia and other without the need of transfusions), or of the co-inheritance of αThal and βThal genes that leads to a less severe unbalancing in α/β chains ratio, or of the HbH disease and of SCD that have remarkable phenotypic heterogeneity related to its underlying molecular defect. Thus immigrants, who have a large number of hemoglobin molecular mutations mirror of their ethnic diversity, considerably increase the complexity of hemoglobinopathies because the wide range of possible interactions and combinations, each with a different phenotype, according to their genetic variability.

School screening with appropriate information and follow-up of discovered carriers is probably the most effective intervention for prospective primary prevention, because sooner carriers are detected and informed, better it is. The same is the case of an immigrant occasionally found affected by microcytic hypochromic anemia but without iron deficiency, especially whether he belongs to ethnic groups with elevated risk for hemoglobinopathies. Other kinds of healthy carrier’s screening should be before marriage(some carriers can avoid risk of hemoglobinopathies selecting a non carrier partner), before conception (a preconception screening is recommended in all at risk couples), during early pregnancy (early prenatal diagnosis in a at-risk couples should be followed by a selective abortion or by a conscious acceptance of a sick child),in foreign groups where consanguineous marriages are common and generally in each immigrant population (this global approach will be possible only after intense programs of information in ethnic minorities).At this regard, a programmatic approach is needed in order not to lose undiagnosed couples at risk. This approach would be offering a carrier screening at the first pregnancy control by adding HPLC to the Rhesus and infectious diseases screening which is performed in most European countries and which should be easily accepted in virtually all the pregnancies.

Finally, a neonatal screening, that places ethical and multicultural-counseling problems (abortion for medical reasons, reassurance, anti-stigmatization), should be offered, as a selective testing, for : 1°) babies of foreign known carriers of a hemoglobin variant; 2°) babies of immigrant parents from an ethnic risk group;3°) babies of at-risk couples with a previous affected child. In Italy, where there is a well developed program for screening at birth for other inherited diseases(phenilketonuria, hypothyroidism, etc.),this hemoglobin screening might be easily added in all immigrant newborns as an *extensive screening*[27]. Obviously, a first primary preventive action of newborn screening for hemoglobinopathies is the incidental detection of babies with clinically insignificant but genetically significant heterozygosity for a variant hemoglobin. In this case it is mandatory to offer educational information and hemoglobin testing both to parents and to whole family. Another important goal of the detection of affected babies by means of neonatal hemoglobinopathy screening is to offer a better management and care from early infancy, reducing morbidity and mortality. Regarding SCD, such better management and care include some vaccinations (against Streptococcus Pneumonia, Haemophilus Influenza type b, meningococcus),administration of prophylactic penicillin, trans-cranial Doppler screening to predict stroke, parental education and support (counseling) as well as transfusions when indicated. In all the other clinically relevant hemoglobinopathies (β thalassemia major, hemoglobin H, combined hemoglobins, etc.)an early diagnosis by neonatal screening enables appropriate blood transfusion regime, adequate iron chelation therapy and narrow transplantation.

Counseling for both healthy carriers and at-risk couples is the last phase of prevention, and cultural elements have to be considered when it is given to individuals with different ethnic origin. The fundamental approaches to multicultural counseling are based on : 1°) *reassurance* (being carries is not an illness, carriers will no develop the disease later, being a carrier is a vantage against malaria); 2°) *anti-stigmatization* (each individual, not only foreigners, can be healthy carrier of hereditary diseases and that can be also the case of either the caregivers or the counselors, abortion for medical reasons is not an immoral and ethically blamable action); 3°) *advantage of knowledge* (known to have a recessive trait can allow pre-marital partner choice, pre-conception prevention of severe affected children in at-risk couples, unnecessary iron therapy in carriers). In addition, counseling provides information regarding the mode of inheritance of hemoglobinopathies, the genetic risk of having affected children and the natural history of hemoglobinopathies, including also their management, care and prevention.

In conclusion, actually Italy is confronting with massive migration movements from developing countries where hemoglobinopathies are widespread. Analogously to other developed countries, this migratory wave is causing a larger diffusion and a changing spectrum in the epidemiology of hemoglobinopathies, constituting an emerging and significant public health problem. In the last years public health authorities of western-northern European and northern American countries have started preventive programs regarding hemoglobinopathies in immigrant populations, whereas in Italy public programs of this kind are yet lacking and the majority of caregivers are yet not aware of this problem due to growing immigration . Therefore, efforts have to be made, offering well organized preventive actions of public health policy. These have to be both prospective and retrospective and based on : 1°) information campaigns and health education programs both for immigrant groups and caregivers (general practitioners, pediatricians, obstetrics, midwifes); 2°) healthy carrier detection among immigrants (school screening, premarital as well as preconception and during early pregnancy screening, screening upon laboratory indications, newborn screening) 3°) proper and multicultural counseling for healthy carriers and for foreign at-risk couples.

## Competing interest

The author declares that he has no competing interests.
